# Outbreak response operations during the US measles epidemic, 2017–19

**DOI:** 10.1186/s12889-021-10652-9

**Published:** 2021-04-13

**Authors:** Elena K. Martin, Matthew P. Shearer, Marc Trotochaud, Jennifer B. Nuzzo

**Affiliations:** Johns Hopkins Center for Health Security and Department of Environmental Health and Engineering, Johns Hopkins University Bloomberg School of Public Health, 621 East Pratt Street, Suite, Baltimore, 210 USA

**Keywords:** Measles, Outbreak response, Public health practice, Immunization

## Abstract

**Background:**

To understand operational challenges involved with responding to US measles outbreaks in 2017–19 and identify applicable lessons in order to inform preparedness and response operations for future outbreaks, particularly with respect to specific operational barriers and recommendations for outbreak responses among insular communities.

**Methods:**

From August 2019 to January 2020, we conducted 11 telephone interviews with 18 participants representing state and local health departments and community health centers that responded to US measles outbreaks in 2017–19, with a focus on outbreaks among insular communities. We conducted qualitative, thematic coding to identify and characterize key operational challenges and lessons identified by the interviewees.

**Results:**

We categorized principal insights into 5 topic areas: scale of the response, vaccination operations, exclusion policies, community engagement, and countering anti-vaccine efforts. These topics address resource-intensive aspects of these outbreak responses, including personnel demands; guidance needed to support response operations and reduce transmission, such as excluding exposed or at-risk individuals from public spaces; operational challenges and barriers to vaccination and other response activities; and effectively engaging and educating affected populations, particularly with respect to insular and vulnerable communities.

**Conclusions:**

Measles outbreak responses are resource intensive, which can quickly overwhelm existing public health capacities. Early and effective coordination with trusted leaders and organizations in affected communities, including to provide vaccination capacity and facilitate community engagement, can promote efficient response operations. The firsthand experiences of public health and healthcare personnel who responded to measles outbreaks, including among insular communities, provide evidence-based operational lessons that can inform future preparedness and response operations for outbreaks of highly transmissible diseases.

**Supplementary Information:**

The online version contains supplementary material available at 10.1186/s12889-021-10652-9.

## Background

The United States is facing a re-emergence of vaccine-preventable diseases, such as measles, particularly among populations with low vaccination coverage [1–5]. These outbreaks will likely continue as anti-vaccine sentiment and vaccine hesitancy increase [6, 7]. The United States eliminated measles in 2000 [8]; however, numerous large outbreaks occurred in multiple states in 2017–19, following imported cases in travelers arriving from countries with active local transmission. Prolonged transmission originating with imported cases nearly resulted in the United States losing its measles elimination status in 2019 [9].

Due to growing skepticism regarding the safety and need for vaccines, reintroductions can lead to outbreaks in communities with pockets of under-vaccination [2, 10]. Measles is highly transmissible, and outbreaks can quickly result in large numbers of cases and contacts, particularly if vaccination coverage is low or following exposures in public spaces (e.g., stores, large gatherings). Rapid spread and numerous contacts and exposures require significant public health resources to contain a measles outbreak, even with an available vaccine.

Recent US outbreaks of measles and other vaccine-preventable diseases have frequently occurred within insular communities, defined as individuals or groups which maintain a degree of separation or isolation from communities or cultures different than their own, including high-profile outbreaks in New York [11, 12], Minnesota [13], Washington [14], and Ohio [15]. Beyond insular communities, pockets of low vaccination coverage fueled a multi-state epidemic in 2015, following exposure to imported cases at Disney theme parks in California [16]. Responding to outbreaks in insular communities can present unique operational challenges, including effective surveillance, vaccination, and community engagement efforts.

This study aimed to identify and understand operational challenges during responses to 2017–19 US measles outbreaks. These outbreaks—ranging in scale from several cases to several hundred—provide an opportunity to characterize the types of barriers to implementing effective response operations that can be expected for highly transmissible disease outbreaks, especially among insular communities. Practical knowledge gained from firsthand outbreak response experience can inform preparedness and response efforts for future outbreaks, particularly in jurisdictions with similar vulnerable populations.

## Methods

We conducted a series of semi-structured, key informant interviews with frontline public health and healthcare professionals who responded to US measles outbreaks in 2017–19. We conducted an initial analysis of peer-reviewed literature and news media to identify outbreaks of interest and prospective interviewees as well as to outline operational barriers and deficiencies to discuss during our interviews. We developed the interview outline (Appendix A) as a starting point, but largely allowed the interviewees to direct the conversation and address priority topics and challenges based on their experience.

We conducted 11 telephone interviews with 18 participants from August 2019 to January 2020. Interviewees represented state and local health departments and healthcare facilities that participated in planning or implementing measles response operations. Their expertise included epidemiology, public health preparedness, community outreach, healthcare administration, and clinical medicine. We included 9 local and 2 state jurisdictions, representing 8 total states, with outbreaks that account for more than 1300 measles cases. All interviews were conducted on a not-for-attribution basis in order to promote transparency, and all interviewees consented to recording interview audio to supplement our interview notes.

We conducted a 2-stage qualitative, thematic coding process on the aggregated interview notes. The initial thematic framework was based on priority research topic areas for Outbreak Observatory [17]—the project under which this study was conducted—and included 8 principal themes: disease surveillance, non-pharmaceutical interventions, medical countermeasures, engaging public health and healthcare, public health resources and infrastructure, public communication and community engagement, community partnerships, and recommendations. Two team members conducted an initial round of coding and identified key topics within each theme and then coded each main theme again to identify more detailed information for each of the key topics [18].

This study was not intended to generate generalizable results, but rather, to capture the scope of challenges, lessons, and recommendations for measles outbreak response operations based on the firsthand experiences of frontline public health and healthcare responders. We encouraged interviewees to discuss topics they felt were most relevant to their experience, and we did not attempt to quantify the prevalence of specific topics in the interviews. Not all topics were addressed in every interview, and the absence of a topic from an interview does not necessarily reflect that the interviewee disagreed with it.

Johns Hopkins Bloomberg School of Public Health Institutional Review Board determined that this study did not constitute human subjects research.

## Results

### Scale of the response

Though contact tracing can be a key component of outbreak responses, highly transmissible diseases that result in many potential exposures can exceed existing contact tracing. Multiple informants indicated that the scale of their measles outbreaks outpaced existing contact tracing capacity. In one jurisdiction, 16 confirmed measles cases yielded more than 3000 contacts (and still increasing at the time of the interview). Several interviewees emphasized the tremendous difference in the scale of their measles response compared to other infectious diseases (eg, mumps), and most discussed needing supplemental personnel to conduct contact tracing and interviews. While several jurisdictions received support from the state health department or neighboring jurisdictions, most relied on local government agencies to provide supplemental personnel. Some jurisdictions had plans in place to collaborate across health department divisions and other local government agencies in order to facilitate utilizing non-public health personnel to support response activities.

Multiple interviewees noted that training supplementary personnel was burdensome. Public health personnel were needed to provide the training, which drew them away from their response duties and further stressed limited public health personnel resources. Additionally, prolonged responses required rotating supplementary personnel into the response in order to mitigate negative effects on routine public health or other government activities, which required further training for new personnel. Numerous participating jurisdictions utilized the Incident Command System (ICS) to organize and conduct their response, and the standardized ICS framework enabled personnel who had completed the required training to plug directly into the response. Several interviewees noted, however, that a lack of familiarity with ICS principles and workflow in their jurisdictions, especially in contrast to routine operations, posed barriers among both public health and supplemental personnel. They emphasized that training and exercising ICS in advance was critical to mitigating this challenge.

### Vaccination

All participating jurisdictions conducted vaccination in some capacity during their response. There was no common approach between jurisdictions, ranging from directing individuals to their healthcare provider to operating community-based vaccination clinics. Most jurisdictions relied on local healthcare providers to administer vaccines, while fewer conducted vaccine campaigns. Uniformly, interviewees described having insufficient personnel to provide vaccinations at a scale necessary to implement a measles outbreak response, so they leveraged local healthcare systems to provide that capacity. Healthcare providers have access to MMR vaccine as part of routine childhood immunizations and the ability to submit for insurance reimbursement, which many health departments may not be capable of doing. Interviewees noted that local providers had the added advantage of pre-established relationships with affected communities, which proved valuable for higher-risk, insular populations that may be more wary of government-affiliated institutions.

Participating jurisdictions that *did* conduct vaccination operations largely served as a safety net for those who were unable to access vaccination through a local healthcare provider. Most administered vaccinations through health clinics or small community events as opposed to large-scale mass vaccination operations (eg, traditional point of dispensing [POD]). Informants noted that collaborating with community partners, including faith leaders and schools, was valuable for holding community events; however, these events were typically small and resulted in few vaccinations. One participating state administered a substantial number of vaccinations, most of which *were* administered at PODs; however, this was for a larger epidemic with outbreaks distributed across multiple local jurisdictions, and mass vaccination efforts were supported by state resources, as opposed to a purely local effort. Multiple interviewees noted that they faced no major barriers to obtaining necessary vaccine doses from state and federal health agencies, and several jurisdictions utilized vaccines provided through the federal Vaccines for Children or Section 317 programs [19].

### Exclusion policies

Due to uncertainties about patients’ infection status, stigma associated with the term’s “isolation” and “quarantine,” and concern about transmission risk to susceptible individuals without an identified exposure, jurisdictions took a variety of actions to keep infectious and susceptible individuals separated. A number of interviewees described these efforts broadly as “exclusion,” which encompassed more traditional isolation and quarantine as well as various modifications. Modified exclusion measures were less restrictive than full or mandatory isolation or quarantine, focusing rather on keeping infectious, exposed, or at-risk individuals out of specific settings (e.g., schools, workplaces). Exclusion policies applied to children and adults without documented measles immunity, either via antibody titers or immunization documentation. Some health departments deliberately selected the term “exclusion” due to negative connotations associated with “quarantine,” and multiple interviewees discussed the challenge of balancing enforcement against encouraging cooperation, wanting to avoid potentially infected individuals going “underground” where health officials could not monitor them. Some jurisdictions did not have pre-existing plans for operationalizing exclusion, and interviewees expressed a need for state or federal guidance, including criteria for determining when individuals could return from exclusion. Each jurisdiction pursued a different mix of exclusion activities depending on local conditions.

Most jurisdictions did have policies in place for removing susceptible children from school, but excluding adults from work, including teachers, was more difficult. State legislation existed regarding vaccination requirements for children to attend school, but similar requirements did not exist for teachers, childcare workers, and other adults. Additionally, childcare centers may not have the same vaccination requirements or oversight as schools, which can facilitate pockets of low coverage. One state-level interviewee discussed that federal funding for schools and childcare programs may stipulate specific attendance requirements for students, and lengthy absences due to exclusion (eg, 21-day incubation period) could jeopardize that funding. This could incentivize schools to enforce vaccination requirements or potentially disincentivize them from excluding exposed, infectious, or susceptible students.

Quarantine was typically reserved for individuals with known high-risk exposure and without documented immunity. Some jurisdictions discussed using “voluntary quarantine” when possible, with mandatory quarantine orders reserved for individuals who did not comply with voluntary provisions. State and local health officials described how court-ordered quarantine was utilized as a back-up measure, if escalated methods of enforcement were required; however, questions remained for public health and law enforcement regarding how to enforce these orders. In some instances, quarantine more closely resembled active monitoring, during which individuals self-monitored for symptoms and reported their condition to health officials.

### Community engagement

Jurisdictions responding to outbreaks concentrated among insular groups required culturally appropriate and sensitive response activities and health messaging to meaningfully engage with affected communities. Some insular communities had long-standing mistrust of government officials, which posed barriers to effective risk communication and promoting appropriate protective action. Interviewees recommended identifying key community leaders, including faith-based and community organizations and healthcare providers, to facilitate engagement with individuals and groups who may be wary of public officials. Several participants commented that developing these relationships during the response was challenging, which complicated efforts to scale up community engagement. Jurisdictions that established relationships in advance of their outbreak discussed being able to more effectively implement outbreak control interventions for higher-risk populations. Jurisdictions that implemented proactive community engagement in advance of their outbreak (eg, regular public forums) found this helpful for identifying and addressing concerns among these communities and establishing trusted relationships. Multiple jurisdictions established relationships with local religious leaders and healthcare providers during previous outbreaks, and several discussed implementing formalized efforts, including regularly scheduled meetings, to strengthen these relationships.

Several interviewees discussed identifying appropriate communication avenues as a principal challenge, including the need to tailor communication strategies for communities that did not use traditional media platforms to share information. Some communities preferred print messaging, and others utilized word-of-mouth communication through social networks and from community leaders rather than traditional or social media platforms typically utilized by local public health agencies. Participants also discussed the importance of being representative and inclusive of the communities they serve. One jurisdiction developed cultural liaison teams to facilitate collaboration between the health department and the community, including to conduct public outreach, craft appropriate messaging, and incorporate culturally sensitive practices into contact tracing and case investigations. In several instances, local faith leaders or healthcare providers joined public health officials during case investigations to help establish trusted relationships and educate the public about culturally appropriate and effective protective actions. One health department emphasized the value of employing community members, which provided insight into community challenges and helped establish comfort between affected individuals and health officials. Adapting engagement plans to communities’ preferences improved uptake of public health messaging and guidance.

These outbreaks garnered substantial media attention, particularly considering the scale of the US measles epidemic, growing vaccine hesitancy and anti-vaccine sentiment, and the involvement of insular communities. While the media enabled health departments to reach a broader audience to communicate about risks (including potential exposure sites) and protective actions, interviewees emphasized the importance of protecting vulnerable populations’ privacy. Interviewees expressed concern that highlighting the risk to certain communities could stigmatize such groups, further increasing their risk and hindering response operations. This was especially challenging when communicating about potential exposures at locations associated with insular populations, including community centers or places of worship. Establishing trust with insular communities was important for reassuring them that response activities were designed to help rather than be punitive.

### Anti-vaccine efforts

Anti-vaccine sentiment contributes to low vaccination coverage that facilitates the re-emergence of diseases like measles and directly impacts response operations. Multiple interviewees, particularly those involved in high-profile measles outbreaks, reported targeted efforts by anti-vaccine advocates from outside their community, which diverted attention and resources from response operations. In one jurisdiction, official health department documents were altered and redistributed in the community to spread disinformation about measles and vaccination. In another jurisdiction, anti-vaccine advocates hosted local events to discuss “vaccine safety,” which provided public platforms for them to spread disinformation. In addition to efforts to influence community perceptions about vaccination, one jurisdiction reported receiving numerous Freedom of Information Act (FOIA) requests and legal challenges to response activities and policies, which drew personnel away from their response duties. Interviewees described these as sophisticated and coordinated campaigns that preyed on vulnerable and isolated communities, largely facilitated through social media platforms that allowed for the rapid spread of inaccurate information to a broad audience. Some of these efforts appeared to be well resourced, to a level that exceeded local jurisdictions’ ability to combat them.

One jurisdiction noted that the immense volume of information available online, including social media, made it challenging for individuals to distinguish factual information from misinformation. Rather than directly countering anti-vaccine campaigns with public health messaging, this jurisdiction provided platforms for local leaders to address their communities directly and share accurate information about the outbreak and vaccination efforts from a trusted voice. Communication tools such as best practices developed by the Mayo Clinic regarding the use of presumptive language [20] and the Tzim Gezint, a public vaccination education document developed collaboratively by a local health department and healthcare coalition [21], were valuable for engaging and educating insular local communities.

## Discussion

The sheer scale of the response to measles outbreaks can stress available resources in myriad ways. Each case can potentially result in dozens of contacts, and extensive contact tracing efforts may be extremely difficult, even for small outbreaks. Community exposures (eg, religious services, retail stores) that involve many potential contacts can be particularly problematic, and it may be impossible to determine exactly who was present, which can result in unidentified chains of transmission. Additionally, exclusion (eg, isolation, quarantine) requires personnel to liaise with affected individuals, including initial contact to clarify exclusion requirements, symptom monitoring, and providing necessary services. Chronic underfunding of public health means that limited resources are available to conduct activities beyond routine operations, and even well-resourced health departments may need additional personnel or funding to support outbreak response on this scale.

Most health departments employ relatively few dedicated staff to support outbreak response operations, and supplementary personnel may be necessary. Government agencies, including other public health divisions, can be excellent sources for supplementary personnel. Training on contact tracing operations is necessary, but these skills can be taught to non-public health personnel. Pulling government personnel away from their regular duties can negatively impact routine activities, so personnel may need to be rotated into the response—although, this increases training requirements. Routine coordination and training across government agencies, including on ICS, provides familiarity with response frameworks and expectations prior to an emergency, which can reduce the need for just-in-time training and enable personnel to more easily assume response roles.

Vaccination is also a key component of measles responses, but mass vaccination may not be an option for all jurisdictions, especially in terms of maintaining sufficient vaccinator capacity. Local healthcare providers, including FQHCs, are excellent resources for vaccinators. Many already have access to vaccines (eg, routine immunizations) and have established relationships in the community, which proves valuable when engaging with insular communities that may be wary of government officials. Leveraging local vaccination capacity can allow health officials to focus limited resources on other aspects of the response while expanding the reach of vaccination efforts.

Pockets of low vaccination coverage, long-standing mistrust of government officials, and stigma by the broader public pose challenges for health officials when engaging insular communities during outbreaks. It is critical to utilize appropriate communication channels and culturally appropriate messaging and interventions. Local community and faith leaders and healthcare providers can serve as entry points for these communities, offer a trusted voice to lend legitimacy to public officials, and identify concerns or barriers in the community. Developing these relationships during a response is resource intensive; therefore, proactive efforts in advance to establish and strengthen critical community relationships can improve crisis response. Additionally, being representative and inclusive of the community can further facilitate positive engagement and trusted relationships, particularly with insular communities.

Trusted relationships with local communities are critical to implementing response operations. Without trust in health officials, individuals may be hesitant to provide honest responses during epidemiological investigations, including on potential contacts or exposure history. Additionally, “exclusion” mechanisms—including removal from school or workplaces, quarantine, or isolation—require trust to promote cooperation by affected individuals. Voluntary exclusion may not provide necessary enforcement mechanisms, and mandatory exclusion orders may be viewed as confrontational. Health officials may need to tailor their exclusion approach to individuals’ unique circumstances; however, applying exclusion consistently can mitigate the risk of singling out specific individuals or communities. The involvement of insular communities can pique the public’s and media’s interest, increasing scrutiny of the response. Balancing transparency and effective risk communication against protecting vulnerable individuals and communities from stigma can be challenging. Maintaining trusted relationships with affected communities, especially insular groups, and providing support for affected individuals can reinforce that exclusion and other response activities are not punitive.

Outbreak responses can face opposition from anti-vaccine advocates, including from outside the jurisdiction, which can hinder response operations. Anti-vaccine sentiment and associated disinformation campaigns have grown in reach and sophistication and can be well organized and well resourced. Increased media attention around large outbreaks, particularly those affecting insular communities, can provide an elevated platform for these groups, which traffic in fear and mistrust to spread false information about vaccine safety. In tightly knit communities, it can be difficult to effectively communicate positive information and engage communities once false narratives take root, particularly if distrust of local authorities already exists. Health departments may not have the resources necessary to combat anti-vaccine efforts directly, so it is critical to leverage trusted relationships with local communities and community leaders to provide positive messaging from trusted voices to counter anti-vaccine propaganda.

We endeavored to include diverse perspectives and generate operational lessons that other jurisdictions can use; however, our study was not without limitations. The communities affected by these measles outbreaks varied with respect to outbreak size, population size and demographics, geographic location, available resources, and myriad other factors. While we selected participants to achieve diversity in these characteristics, our study population is not necessarily representative of every jurisdiction. Additionally, our study aimed to identify the types of operational challenges that jurisdictions can expect in responding to measles outbreaks, particularly those among insular communities. These lessons may not be wholly generalizable to all outbreaks or communities; however, they provide information to help other jurisdictions anticipate and mitigate these challenges rather than learning these lessons themselves in the midst of a response. Finally, we were not able to include all outbreaks that occurred during 2017–19. Some outbreaks were very small, and not all were publicly reported. Some health departments declined to participate, but for some outbreaks, we identified alternative interviewees to discuss the response, including local healthcare providers. Ultimately, we included outbreaks that account for more than 75% of all US measles cases reported in 2017–19 recognizing this is not inclusive of all jurisdictions reporting measles outbreaks at that time [22].

## Conclusion

Outbreak responses, particularly for highly transmissible diseases like measles, are resource-intensive operations. Personnel limitations can be a barrier for implementing critical operations like contact tracing and vaccination. Many jurisdictions may not have sufficient financial and personnel resources to address these challenges. Local governments and health systems can provide supplementary personnel to support response activities, such as contact tracing and vaccination. Outbreaks among insular communities may require additional resources to facilitate effective engagement, particularly considering increased media interest and anti-vaccine activity. It is critical to establish and leverage trusted relationships with affected communities—including with faith and local community leaders, healthcare providers, media, and the broader public—to implement effective engagement with high-risk populations, particularly in insular communities. These firsthand experiences can help other jurisdictions anticipate potential operational challenges in advance of future outbreaks and incorporate lessons into their own preparedness efforts.

## Supplementary Information

**Additional file 1.** Appendix A: Semi-Structured Interview Script.

## Data Availability

Not applicable.

## Abbreviations

US: United States
ICS: Incident Command System
MMR: Measles Mumps Rubella Vaccine
POD: Point of Dispensing
FOIA: Freedom of Information Act
FQHC: Federally Qualified Health Center
