# Supplementary material for: Outbreak response operations during the US measles epidemic, 2017–19
Source: BMC Public Health. 2021 Apr 13;21:620. doi: 10.1186/s12889-021-10652-9 (PMC8042853; doi:10.1186/s12889-021-10652-9)
Supplement: Supplementary file 1 — Additional file 1. Appendix A: Semi-Structured Interview Script. [file 12889_2021_10652_MOESM1_ESM.docx]

# Appendix A: Semi-Structured Interview Script

General/Introductory Questions

- - Tell us about the measles outbreak your community experienced/is experiencing.
    - What is/was your role in the response?
    - Is this outbreak different from prior measles cases or outbreaks in your jurisdiction? In what ways?
  - Who has primarily been affected by the outbreak?
    - For Example: demographic information, specific subpopulations or groups
    - How many cases were/have been reported? How many hospitalizations?
  - What is the impact of the outbreak on your community?

Response Organization and Activities

- - What actions did your health department undertake to control the outbreak?
    - For Example: contact tracing, vaccination, risk communication?
  - How did you identify people who were potentially exposed, and how wide of a net did you cast?
  - What divisions or programs in your health department, participated in outbreak response activities?
    - For Example: infectious or communicable diseases, epidemiology, public health or private laboratories, immunization?
  - What challenges did you face in conducting outbreak response operations?
    - Did you experience any challenges engaging high-risk or vulnerable populations?
    - What strategies did you implement to combat these challenges?
    - How effective were these strategies in mitigating these challenges?
  - Did the measles outbreak impact other health department operations or programs? How so?
    - Did the outbreak have any negative impact on other health department services?
  - Did your jurisdiction or state declare a public health emergency in response to the measles outbreak?
    - How did this impact outbreak response operations?
  - How well did existing preparedness plans, programs, and funding mechanisms function during the response?

Vaccination Operations

- - What was your agency’s strategy for vaccination during the outbreak?
    - How did you determine where to conduct vaccination operations?
    - How did you determine who would be eligible for vaccination?
  - What challenges did you face with respect to vaccination operations?
  - How did you fund vaccination operations?
  - Did your jurisdiction purchase vaccine to support response activities specifically for this outbreak?
    - From where/whom did you purchase the vaccine?
    - How were these purchases financed?
  - Did you have sufficient doses of vaccine to support response operations?

Risk Communication and Community Outreach

- - We are not focusing specifically on risk communication or public education efforts, because we know that others are already investigating this aspect of outbreak response. But we know that public communication is a critical component to implementing successful outbreak response operations. In that context, what communication challenges did you face, and how did they impact your response activities?
    - What solutions did you implement to mitigate these effects, and how well did they work?
    - What were your highest priority communication challenges?
    - Did vaccine hesitancy or anti-vaccine sentiment influence your response?
    - What kinds of communication resources were valuable to you during your response?
    - What advice would you have for other health jurisdictions about communicating during measles outbreaks?

Community Partnerships

- - Did your health department coordinate with any external partners to support outbreak response operations?
    - For Example: community groups, health systems, nongovernmental organizations (NGOs), Medical Reserve Corps (MRC)
    - What did these groups bring to the response?
    - How well did these partnerships work?

Public Health Resources and Infrastructure

- - Did you experience any resource shortcomings during the response?
    - For Example: financial, personnel, material
  - How did these shortcomings impact response activities?
    - What actions did you take to mitigate these effects?
  - What additional resources do you wish you would have had available in advance of the outbreak?
    - For Example: To improve preparedness or to facilitate response operations
    - How would these resources have better prepared your jurisdiction for a measles other similar outbreaks?
  - Can you estimate the cost of the measles outbreak response in your jurisdiction?
    - Including personnel time, vaccine purchases, and other physical resources
    - Did resource limitations associated with the outbreak negatively impact other health department operations? Can you discuss the magnitude of these effects?

Barriers, Lessons, and Other

- - What was the biggest barrier to containing the outbreak?
  - What would have made your response easier? More difficult?
  - What lessons would you want to share with other impacted communities or jurisdictions planning for future outbreaks of measles or other vaccine-preventable diseases?
  - What is the most surprising thing you learned during the response?
  - Have any other health departments contacted you about your response?
    - For Example: To seek advice about how to respond or how to plan for a potential outbreak
  - Is there anyone else we should talk to, either in your department or jurisdiction, in other neighboring jurisdictions, or at the state or federal level?
